# Invariant NKT Cells Regulate the CD8 T Cell Response during Theiler's Virus Infection

**DOI:** 10.1371/journal.pone.0087717

**Published:** 2014-01-30

**Authors:** Lennart T. Mars, Magali Mas, Lucie Beaudoin, Jan Bauer, Maria Leite-de-Moraes, Agnès Lehuen, Jean-Francois Bureau, Roland S. Liblau

**Affiliations:** 1 Institut National de la Santé et de la Recherche Médicale (INSERM). Unité Mixte de Recherche (UMR)-1043, Toulouse, France; 2 Centre National de la Recherche Scientifique (CNRS), UMR 5282, Toulouse, France; 3 Université de Toulouse, UPS, Centre de Physiopathologie de Toulouse Purpan (CPTP), Toulouse, France; 4 Institut Cochin, INSERM UMR-1016, CNRS UMR 8104, University Paris-Decartes, Paris, France; 5 Center for Brain Research, Department Neuroimmunology, Medical University of Vienna, Vienna, Austria; 6 CNRS-UMR 8147, Université Paris Descartes, Sorbonne Paris Cité, France, Institut Necker Enfants Malades, Paris, France; 7 Unité Génétique Fonctionnelle des Maladies Infectieuses, Institut Pasteur, Paris, France; San Raffaele Scientific Institute, Italy

## Abstract

Invariant NKT cells are innate lymphocytes with a broad tissue distribution. Here we demonstrate that iNKT cells reside in the central nervous system (CNS) in the absence of inflammation. Their presence in the CNS dramatically augments following inoculation of C57Bl/6 mice with the neurotropic Theiler's murine encephalomyelitis virus (TMEV). At the peak of inflammation the cellular infiltrate comprises 45 000 iNKT cells for 1 250 CD8 T cells specific for the immunodominant TMEV epitope. To study the interaction between these two T cell subsets, we infected both iNKT cell deficient Jα18^-/-^ mice and iNKT cell enriched Vα14 transgenic mice with TMEV. The CD8 T cell response readily cleared TMEV infection in the iNKT cell deficient mice. However, in the iNKT cell enriched mice TMEV infection persisted and was associated with significant mortality. This was caused by the inhibition of the CD8 T cell response in the cervical lymph nodes and spleen after T cell priming. Taken together we demonstrate that iNKT cells reside in the CNS in the absence of inflammation and that their enrichment is associated with the inhibition of the anti-viral CD8 T cell response and an augmented mortality during acute encephalomyelitis.

## Introduction

The central nervous system (CNS) is classically considered an immune privileged environment in which immunogens can persist in the parenchyma without eliciting an adaptive immune response [1]–[3]. Despite this feature the CNS is not deprived from immune intervention. Resident glial cells constitute a local innate immune system. Microglia are derived from primitive macrophages and function in the CNS as immune sentinels that search for microbial presence by protruding their processes throughout the surrounding tissue [4], [5]. Focal brain injury attracts microglial processes to the site of insult within minutes [6], [7]. Presence of pathogens or tissue damage will activate the inflammasome of microglia and/or astrocytes via the engagement of Toll Like receptors (TLRs) or nucleotide-binding domain leucine-rich repeat-containing receptors (NLRs) that recognise pathogen-associated molecular patterns (PAMPs) or damage-associated molecular patterns (DAMPS) [8]. This activation of microglia permits the expression of major histocompatibility (MHC) class I and II molecules. This allows microglial cells to present phagocytosed antigen to effector T cells that can efficiently infiltrate the CNS parenchyma after their activation in the secondary lymphoid organs [9]. In the absence of dedicated lymphoid drainage for the CNS the priming of the adaptive immune response is ensured when CNS-tropic pathogens passage through peripheral organs prior to ingress of the CNS [8].

Infections of the CNS usually generate efficient inflammatory responses. This results in the eradication of the infectious agent with limited tissue damage. Occasionally excessive immune activity can cause severe CNS pathology [10], or the infectious agent can escape immune clearance and establish persistence or latency within the parenchyma [11]. Upon CNS inflammation the resident cells cooperate with incoming immune cells to ensure efficient intervention. Activated microglia and probably reactive astrocytes can express MHC class II enabling the presentation of antigen to CD4^+^ T cell responses [12], [13]. Furthermore, MHC class I expression can be induced on all CNS resident cells. Consequently, cytotoxic CD8^+^ T cells are able to kill neurons, oligodendrocytes and astrocytes in an antigen-specific manner that involves the formation of an immunological synapse between neural cells and killer T cells [14]–[17].

The contribution of other T cells to neuroinflammation is less well characterised. In this study we addressed the role of invariant NKT (iNKT) cells during CNS viral infection. iNKT cells express a unique semi-invariant αβ T cell receptor (TCR). In humans, this TCR comprises a Vα24-Jα18 rearranged chain combined with a Vβ11 chain, while in mice this TCR comprises a Vα14-Jα18 chain paired with a restricted set of β-chains [18]. iNKT cells develop in the thymus where CD1d expressing double-positive thymocytes trigger their selection and differentiation [19]–[21]. By virtue of their semi-variant TCR iNKT cells recognise glycolipids that are presented in the context of the non-classical MHC molecule CD1d [22]. The restricted TCR usage by iNKT cells suggests the recognition of a conserved set of antigens. These include lipids produced by bacteria or isolated from house dust indicating that the presence of non-self lipids can mobilise iNKT cells during microbial infections and possibly asthma [23]–[28]. The foreign nature of the lipids is in part reflected by the alpha-anomeric orientation of the glycosidic linkage between the carbohydrate headgroup and the lipid backbone, which is not known to be generated in mammals [29]. During viral infections, autoimmune inflammation or cancer iNKT cells are also activated, probably in response to the presentation of endogenous antigens. These include ligands such as peroxisome-derived lipids [30], Lyso-phospholipids [31], and in mice isoglobotrihexosyl ceramide (iGb3) [21]. The presentation of these endogenous ligands can be induced after activation of the innate immune system by TLR-9 engagement in the presence of type-I interferons [32]. Alternatively, in infected cells, modified self-lipids can be generated by virus-induced secretory phospholipases and serve as antigens for the iNKT cell response [33]. Hence, during microbial infections iNKT cells can be activated by exogenous as well as endogenous lipid moieties.

The functional contribution of iNKT cells to the immune response is highly variable. During infections iNKT cells operate at the interface of innate and adaptive immunity playing a central role in promoting the acquired immune response [18]. Studies regarding their role in organ-specific autoimmunity have revealed an immunosuppressive function for iNKT cells in animal models of type I diabetes and multiple sclerosis [34]. As such, autoimmune inflammation of the CNS can be prevented by the activation of iNKT cells with exogenous glycolipid antigens, or by increasing the frequency of iNKT cells, as is the case in mice that express a transgene encoding the invariant TCR alpha chain [35]–[40]. Less is known regarding the role of iNKT cells during viral infections of the CNS. Theiler's murine encephalomyelitis virus (TMEV) is a neurotropic picornavirus whose natural host is the mouse. In experimental conditions, after intracranial inoculation, this virus not only induces an acute encephalomyelitis in all laboratory strains of mice but is able to persist indefinitely in the white matter of susceptible strains [41]–[43]. In this study we addressed the function of iNKT cells in the resistant C57BL/6 mice, which clear TMEV infection after acute encephalomyelitis.

## Materials and Methods

### Ethics Statement

Animals were housed in the animal facilities of the Pasteur Institute (accreditation number 75-15-01) or the INSERM (Zootechnie UMS-006; accreditation number A-31 55508) that are accredited by the French Ministry of Agriculture to perform experiments on live mice in appliance of the French and European regulations on care and protection of the Laboratory Animals (EC Directive 2010/63, French Law 2013-118, February 6th, 2013). All protocols using TMEV infection were approved by the veterinary staff of the Pasteur Institute animal facility which has been accredited with an NIH Animal Welfare Insurance #A5476-01 issued on 31/07/2012. Non-infectious experiments were performed in the INSERM animal facility (Zootechnie UMS-006) under protocol (04 U563RL-27) and approved by the facilities veterinary staff and the Midi-Pyrénées ethics committee (approved by the ministry of higher education and research) in accordance to the above-mentioned European regulations. Animals were housed under standard conditions with a 12-hour light/dark cycle and access to food and water *ad libitum*. Animal suffering was reduced by keeping the number of animals used to a minimum required for the planned studies to give statistically significant data.

### Animals

The Jα18^-/-^ and Vα14-Jα18 transgenic (Vα14 Tg) non-obese diabetic (NOD) mice have already been described [44], [45]. The Vα14 Tg NOD mice were crossed on a C57Bl/6 background for over 12 generations to create the Vα14 Tg C57Bl/6 mouse line. These mice were bred and maintained under specific pathogen free (SPF) conditions and used with sex-matched controls between 4–8 weeks of age.

### TMEV, Peptides, and Infection

The Daniels (DA) strain of TMEV, was produced by transfection of BHK-21 cells with the pTM762 plasmid as described elsewhere [46], [47]. TCR Vα14-Jα18 Tg and Jα18^-/-^ mice on the resistant C57Bl/6 background were anaesthetized with ether and infected intracerebrally with 10^4^ PFU of TMEV in 40 µl of phosphate-buffer saline.

The H2-D^b^ restricted VP2 derived peptide 122–130 (HAGSLLVFM) was synthesized by Neosystem (Strasbourg, France) to a purity of >90%.

### CNS-infiltrating mononuclear cell purification

Anesthetized mice were perfused through the left cardiac ventricle with cold PBS. The brain and spinal cord were dissected. Brain tissue was dissociated by passages over three consecutive cell-strainers of 150, 100, 40 microns. Single-cell suspensions were resuspended in 30% percoll and deposited on 70% percoll. After a 20-minute centrifugation at 3000 rpm mononuclear cells were collected at the interface, washed twice in medium and used for further analysis.

### ELISpot

Peptide-specific T cells were enumerated using an ELISpot assay as previously described [48]. 96 well plates containing nitrocellulose filters (Multiscreen; Millipore Corp., Molsheim, France) were coated overnight with sterile anti-IFN-γ mAbs at 4°C (R4-6A2, BD Biosciences). The plates were washed 3 times with DMEM and saturated for one hour with complete DMEM. Single cell splenocytes or Percoll purified mononuclear cells from the CNS, were cultured at 1×10^6^ cells/well and restimulated or not with the VP2_122–130_ peptide at 1 or 10 µM (triplicates). After 40 hrs, plates were washed three times with PBS-Tween and PBS, incubated 2 hrs with biotinylated anti-mouse IFN-γ (XMG1.2, BD Biosciences) and 1.5 hrs with alkaline phosphatase–conjugated streptavidin (Roche Molecular Biochemicals, Mannheim, Germany). Spots were developed by adding phosphatase substrates, 5-bromo-4,3-indolyl phosphate, and nitroblue tetrazolium (Promega Corp., Madison, Wisconsin, USA) and spot forming cells (SFC) counted using the automated image analysis system KS ELIspot (Zeiss, Le Pecq, France). A response >10 spots over background per well was considered specific. Every mononuclear cell sample was analyzed by flow cytometry to assess the proportion of CD8^+^TCRβ^+^ T cells. This proportion was used as denominator for the frequency of DA-peptide specific CD8 T cells. The absolute number was calculated based by multiplying the frequency of SFC with the total number of mononuclear cells obtained after percoll purification.

### RNA isolation

Isolated cells were resuspended in RNAprotect cell reagent and kept at −80°C until use. RNA was extracted using a RNeasy-plus mini kit and QiaShredders according to the manufacturer's instructions (Qiagen, Courtaboeuf, France). The quality of the RNA was assessed by capillary electrophoresis (Agilent Bioanalyzer, Agilent Technologies) and by spectrometry (λ260/λ280) prior to further use. One µg of total RNA was converted into a representative cDNA pool using the Superscript III First-Strand Synthesis System and random-hexamer primers, according to the manufacturer's instructions (Invitrogen, Cergy Pontoise, France).

### QPCR primers and gene-expression analysis

The following published primer pairs were used: CCL5: forward: 5′-GCCCACGTCAAGGAGTATTTCTA-3′, reverse: 5′-ACACACTTGGCGGTTCCTTC-3′; CXCL10: forward 5′-GCCGTCATTTTCTGCCTCAT-3′, reverse 5′-GCTTCCCTATGGCCCTCATT-3′; GATA-3: forward 5′-CTTATCAAGCCCAAGCGAAG-3′, reverse 5′- CCCATTAGCGTTCCTCCTC-3′; Granzyme B: forward 5′-CGATCAAGGATCAGCAGCCT-3′, reverse 5′-CTTGCTGGGTCTTCTCCTGTTCT-3′; HPRT: forward 5′-TGACACTGGTAAAACAATGCAAACT-3′, reverse 5′-AACAAAGTCTGGCCTGTATCCAA-3′; IFN-γ: forward 5′-TCAAGTGGCATAGATGTGGAAGAA-3′, reverse 5′-TGGCTCTGCAGGATTTTCATG-3′; IL-4: forward 5′-ACAGGAGAAGGGACGCCAT-3′, reverse 5′-GAAGCCCTACAGACGAGCTCA-3′; IL-10: forward 5′-GGTTGCCAAGCCTTATCGGA-3′, reverse 5′-CTGTCATCGATTTCTCCC-3′; IL-13: forward 5′-GGAGCTGAGCAACATCACACA-3′, reverse 5′-GGTCCTGTAGATGGCATTGCA-3′ IL-17: forward 5′-CCACGTCACCCTGGAACTCTC-3′, reverse 5′-CTCCGCATTGACACAGCG-3′.

Perforin: forward 5′-GAAGACCTATCAGGACCAGTACAACTT-3′, reverse: 5′-CAAGGTGGAGTGGAGGTTTTTG-3′; Rorγt: Forward 5′- TTCACCCCACCTCCACTG-3′, reverse 5′-CAAGGGATCACTTCAATTTGTG-3′; TNF-α: forward 5′-CATCTTCTCAAAATTCGAGTGACAA-3′, reverse 5′-CACGTCGTAGCAAACCACCAAGTGGA-3′; T-bet: forward 5′-TCAACCAGCACCAGACAGAG-3′, reverse 5′-AAACATCCTGTAATGGCTTGTG-3′.

The housekeeping gene HPRT was used as an endogenous control. Quantitative PCR was performed using the ABI Prism 7000 Sequence Detection System (Applied Biosystems, Foster City, USA). Each reaction was performed in 25 µl volume in a 96-well plate (Applied Biosystems) containing 1 µl of cDNA, 12.5 µl of SYBR Green I Master Mix 2X (Eurogentec, Angers, France), 30 nM of yeast tRNA (Sigma), and 300 nM each of the forward and reverse primers (Invitrogen). Conditions for the PCR were 2 min at 95°C, and then 40 cycles, each consisting of 15 s at 95°C and 1 min at 60°–62°C.

The ΔΔCt calculation for the relative transcript quantification was applied as follows ΔΔCt  =  (Cttarget gene – CtHPRT)x – (Cttarget gene – CtHPRT)y, where x  =  sample to analyze and y  =  an arbitrarily chosen control sample. Results for each sample were expressed as fold changes in target gene copies, according to the following equation: amount of target transcripts  =  2-ΔΔCt. Two independent experiments were carried out for each gene and sample. In each experiment, each sample was tested in duplicate wells.

### Viral load determination

Quantification of viral genome was performed by real-time PCR as described previously [49] using as primers HPRT: Forward CTGGTGAAAAGGACCTCTCG, Reverse TGAAGTACTCATTATAGTCAAGGGCA and probe Yakima Yellow-TGTTGGATACAGGCCAGACTTTGTTGGAT-DDQ1; TMEV : Forward GCCGCTCTTCACACCCAT, Reverse AGCAGGGCAGAAAGCATCAC and the probe 6FAM-CGACGTGGTTGGAGAT-DDQ1. Viral load is presented as the square root of the TMEV copy-number over the copy-number of HPRT.

### Flow cytometry

After Fcγ receptor blockade with the 2.4G2 mAb, cells were stained with biotinylated CD1d:α-GalCer tetramers labeled with allophycocyanin, as previously described [50]. Additional stainings were performed with the following mAbs: anti-β TCR (H57-597), anti-CD4 (RM4-5), anti-CD8 (53.6.2). Cells were acquired with a FACSCalibur (Becton Dickinson, Mountain View, CA) and analyzed using CellQuest or Flowjo software. For cell-sorting, cells were passed through a FACSAria flow-cytometer (Becton Dickinson, Mountain View, CA) using FACS-diva software.

### Histology

For neuropathology, deeply anesthetized mice were transcardially perfused with 4% paraformaldehyde. Tissues were embedded in paraffin. Immunohistochemistry was performed without pretreatment as previously described [51], using a Rabbit Polyclonal serum (1∶2500) produced against purified TMEV virus, with haematoxylin as nuclear counterstain [52]. Labeling was visualized with 3,3 diaminobenzidine-tetra-hydrochloride (Sigma-Aldrich).

### Statistical Analysis

Statistical significance was determined using the two tailed non-parametric Mann-Whitney U test and P values below 0.05 were accepted as significant (*p<0.05; **, p<0.01; ***, p<0.001).

## Results

### Invariant NKT cells reside in the CNS in the absence of inflammation

**Figure 1:**
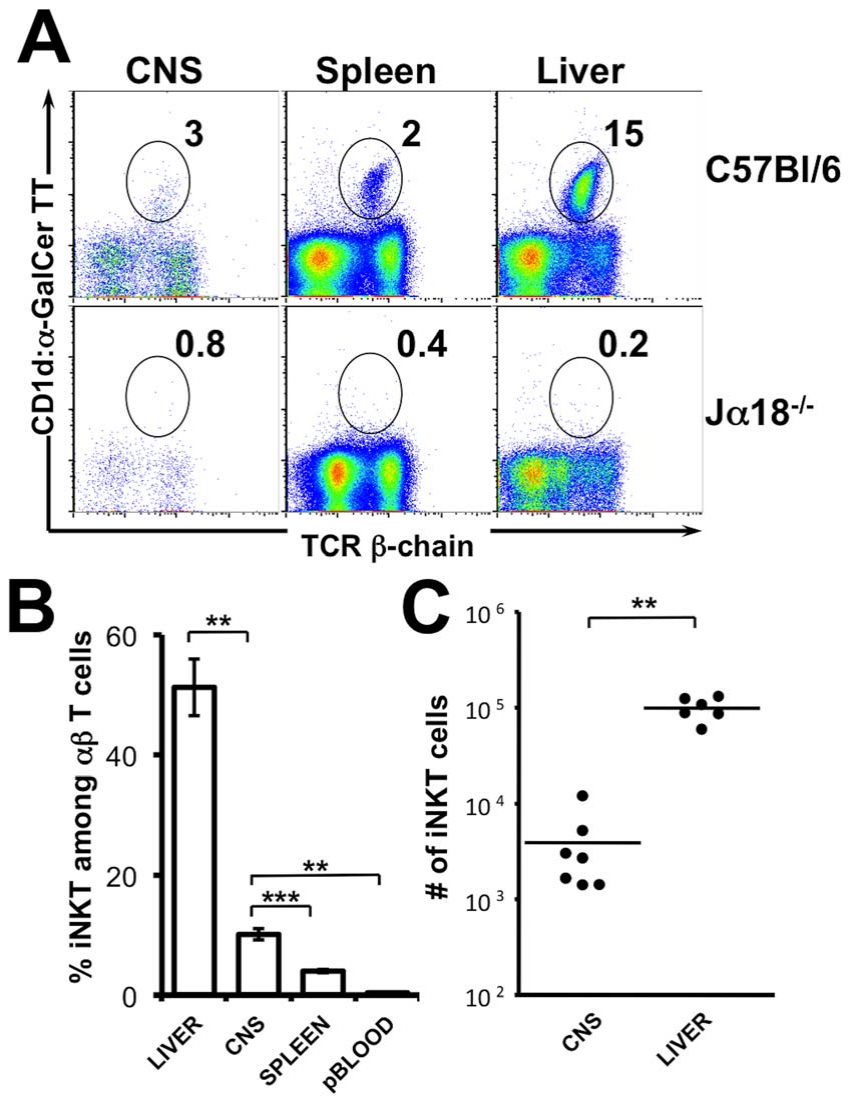
iNKT cells reside in the CNS in the absence of inflammation. (A) Mononuclear cells were isolated from the brain and spinal cord (left), spleen (middle), and liver (right) of unmanipulated adult C57Bl/6 (top) or Jα18^-/-^ C57Bl/6 mice (bottom) by percoll gradient. A representative CD1d:α-GalCer tetramer staining on mononuclear cells is presented. (B) Frequency of CD1d:α-GalCer tetramer positive cells among αβ T cells in the liver (n = 6), spleen (n = 7), peripheral blood (n = 5), CNS (n = 7, 3–16 pooled tissues/observation). (C) Absolute numbers of CD1d:α-GalCer tetramer positive αβ T cells in the liver (n = 5) and CNS (n = 7, 3–16 pooled tissues/observation) among percoll purified mononuclear cells. Data represent the mean ± SEM of n individual mice per group.

iNKT cells are widely distributed throughout the body. They express an effector/memory phenotype associated with a chemokine/homing receptor profile affording their presence in non-lymphoid tissues [53]–[55]. To test if iNKT cells could reside in the CNS in the absence of inflammation we assessed the frequency of CD1d:αGalCer positive iNKT cells among mononuclear cells purified from uninfected wild-type animals. As shown in figure 1, the CD1d:αGalCer tetramer provided little or no staining of mononuclear cells purified from the CNS or liver of TcR Jα18^-/-^ mice that selectively lack iNKT cells (Fig 1A). By contrast, in C57Bl/6 wild-type mice iNKT cells represented, on average, 10% of total T cells isolated from the CNS (Fig 1B). To reduce contamination by circulating iNKT cells all mice were transcardially perfused with PBS prior to the isolation of CNS mononuclear cells. Moreover, iNKT cells in the peripheral blood, taken prior to PBS perfusion, only made up for 0.3% of circulating T cells. The frequency of iNKT cells in the CNS is therefore, significantly higher than that detected among T cells in the peripheral blood (Fig 1B). Taken together, these arguments strongly suggest that iNKT cells reside in the CNS even in the absence of inflammation. By multiplying the frequency of iNKT cells with the total number of mononuclear cells obtained after percoll purification, we estimated the absolute number of iNKT cells to amount to an average of 3900 iNKT cells per CNS (Fig 1C).

### Invariant NKT cells infiltrate the CNS after local viral infection

**Figure 2:**
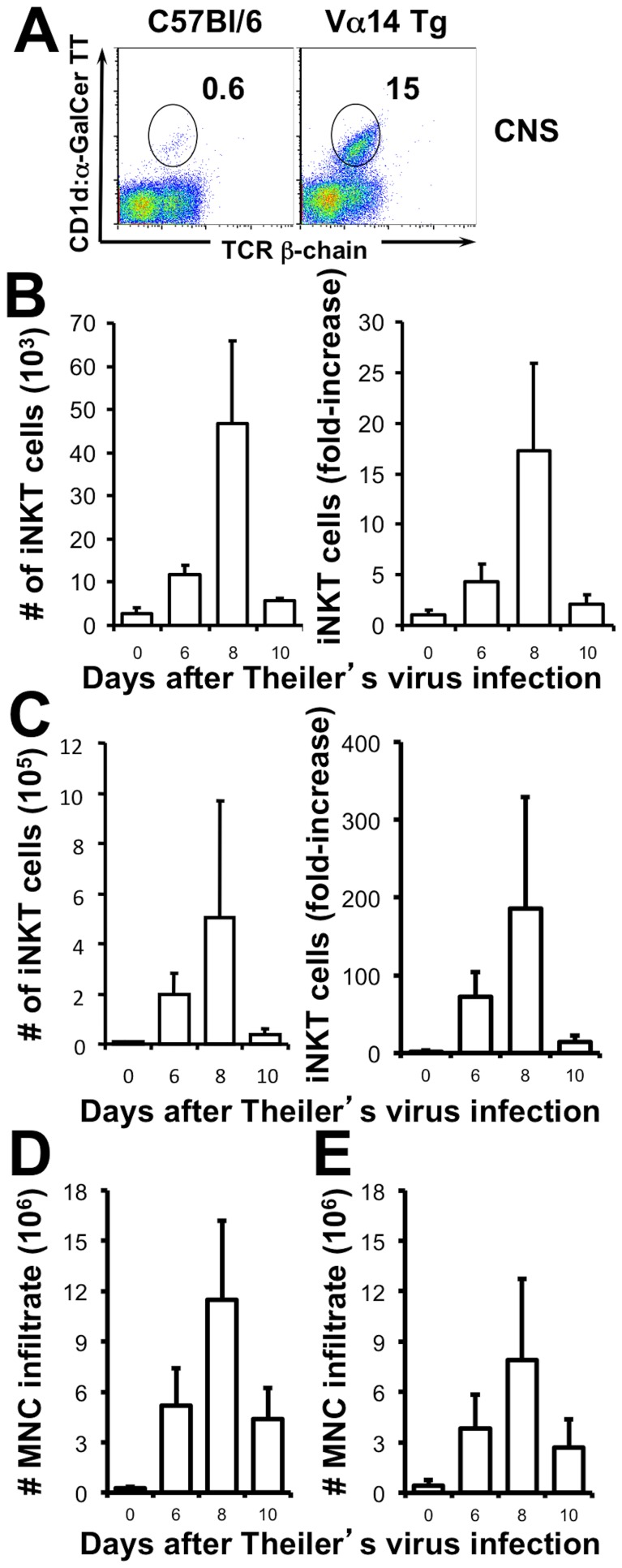
iNKT cells accumulate in the CNS during TMEV infection. (A) Representative CD1d:α-GalCer tetramer staining on purified mononuclear cells from the CNS of wild type C57Bl/6 (left) and TCR Vα14 Tg mice (right) 8 days after infection with 10^4^ pfu of TMEV. (B–C) Absolute number of CD1d:α-GalCer αβ T cells (left) and their fold increase (right) in the CNS after TMEV infection of C57Bl/6 mice (B) and Vα14 Tg mice (C). Absolute number of purified mononuclear cells from the CNS of C57Bl/6 (D) and Vα14 Tg mice (E). Data represent the mean ± SEM of 2 independent experiments with 2–4 pooled mice/observation.

Following intracerebral inoculation with the DA strain of TMEV (10^4^ pfu/mouse) the virus initially replicates in neurons and all mice develop acute grey matter encephalitis. In resistant mouse strains such as the C57Bl/6, this infection is cleared within 3 weeks by an anti-viral CD8 T cell response peaking 8–10 days post-inoculation (p.i.). To assess if this adaptive immune response is accompanied, or even preceded, by the infiltration of iNKT cells in the CNS we infected C57Bl/6 mice and TCR Vα14 transgenic (Vα14 Tg) mice with TMEV and assessed the absolute numbers of iNKT cells that infiltrated the CNS at different timepoints p.i.. As shown in figure 2A, iNKT cells were identified among percoll purified mononuclear cells from the CNS using CD1d:αGalCer tetramers. In TMEV infected wild-type mice an average of 4.5×10^4^ iNKT cells could be detected at the peak of inflammation (8 days p.i.) representing a 17-fold amplification over basal levels (Fig 2B). This was accompanied by a massive infiltration of mononuclear cells to the CNS (Fig 2D) that diluted the overall frequency of iNKT cells among the inflammatory infiltrate. In the iNKT cell-enriched Vα14 Tg mice the intracerebral infection with TMEV caused an accumulation of iNKT cells with similar dynamics. At the peak of inflammation an average of 50×10^4^ iNKT cells had accumulated in the CNS, representing a 200-fold increase over basal levels (Fig 2C). Similar to the wild-type mice, infection of the Vα14 Tg mice caused strong local inflammation (Fig 2E). These observations indicate that iNKT cells accumulate locally during CNS viral infection.

### CNS viral infection is lethal in mice enriched in iNKT cells

**Figure 3:**
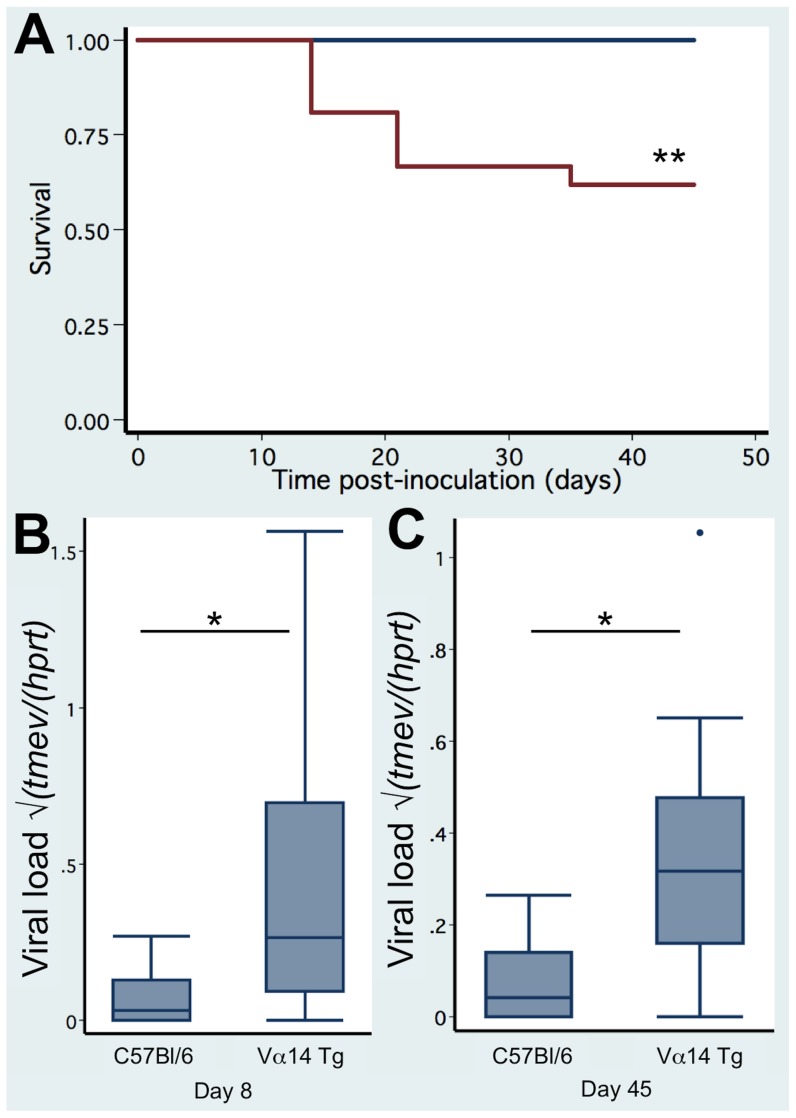
Increased mortality and viral load in TCR Vα14 Tg mice infected with TMEV. (A) Kaplan-Meyer survival curve of Vα14 Tg C57Bl/6 mice (n = 21) and C57Bl/6 littermates (n = 19) infected with 10^4^ pfu of TMEV. (B) TMEV viral load in the spinal cord 8 days after infection of Vα14 Tg C57Bl/6 mice (n = 13) and C57Bl/6 littermates (n = 19). (C) TMEV viral load in the spinal cord 45 days after infection of Vα14 Tg C57Bl/6 mice (n = 19) and C57Bl/6 littermates (n = 8). Data represent the pooled observations of 2–3 independent experiments.

**Figure 4:**
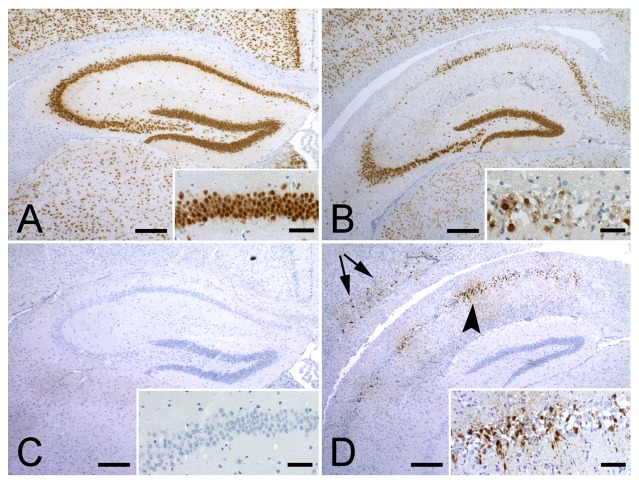
Neuropathology of TMEV infection. (A) In C57Bl/6 mice NeuN staining demonstrated no degeneration in the hippocampus 12 days post-infection with TMEV. Inset shows a higher magnification of the CA1 region. (B) In Vα14 Tg C57Bl/6 mice NeuN staining revealed neuronal loss in CA1 and CA2 regions of the hippocampus 12 days post-infection with TMEV. (C) On consecutive sections, TMEV protein could not be detected in C57Bl/6 mice, while in the Vα14 Tg C57Bl/6 mice (D) TMEV was readily detectable in the hippocampus (arrowhead) and in the cortex (arrows). Insets show higher magnifications of the CA1 region.

The clinical consequence of neurotropic viral infection was assessed in Vα14 Tg mice, Jα18^-/-^ mice, and C57Bl/6 wild-type mice over a period of 45 days. As shown in Figure 3A, 8 out of 21 (38%) Vα14 Tg mice that are enriched in iNKT cells died. In contrast, none out of the 19 C57Bl/6 littermates died (LogRank test p = 0.003). The increased mortality was correlated with a significantly augmented viral load in the Vα14 Tg mice that was detectable at day 8 (0.40±0.09 (n = 19) versus 0.07±0.03 (n = 8) in C57Bl/6; p = 0.04) and persisted until day 45 (0.36±0.10 (n = 13) versus 0.07±0.02 (n = 19) in C57Bl/6; p = 0.0002) (Fig 3 B–C). Histological analysis of infected Vα14 Tg mice revealed presence of TMEV (Fig 4D) with coinciding neuronal degeneration (Fig 4B) in the hippocampus and cortex 12 days after TMEV infection (2 out of 3 mice analysed). No degeneration or TMEV was detected in a single wild type mouse at the same time point (Fig 4A and C). By day 35 post infection, TMEV was no longer detected by immunohistochemistry in Vα14 Tg mice (0 out of 7 mice) or wild-type mice (0 out of 6 mice), only in H2-D^b -/-^ + H2-K^b -/-^ that lack a CD8 T cell compartment could we detect presence of TMEV in the hippocampus and cortex (4 out of 8 mice, data not shown). The wild-type and Jα18^-/-^ mice had similar very low viral load in the CNS at 8 and 45 days post infection (data not shown). These observations indicated that the enrichment in iNKT cells permitted a persistent infection of TMEV, which was correlated with an increased mortality. Given the striking phenotype in the Vα14 Tg mice we focussed our study on the comparison between TMEV infected Vα14 Tg mice and C57Bl/6 mice.

### The enrichment in iNKT cells inhibits the anti-viral CD8 T cell response

**Figure 5:**
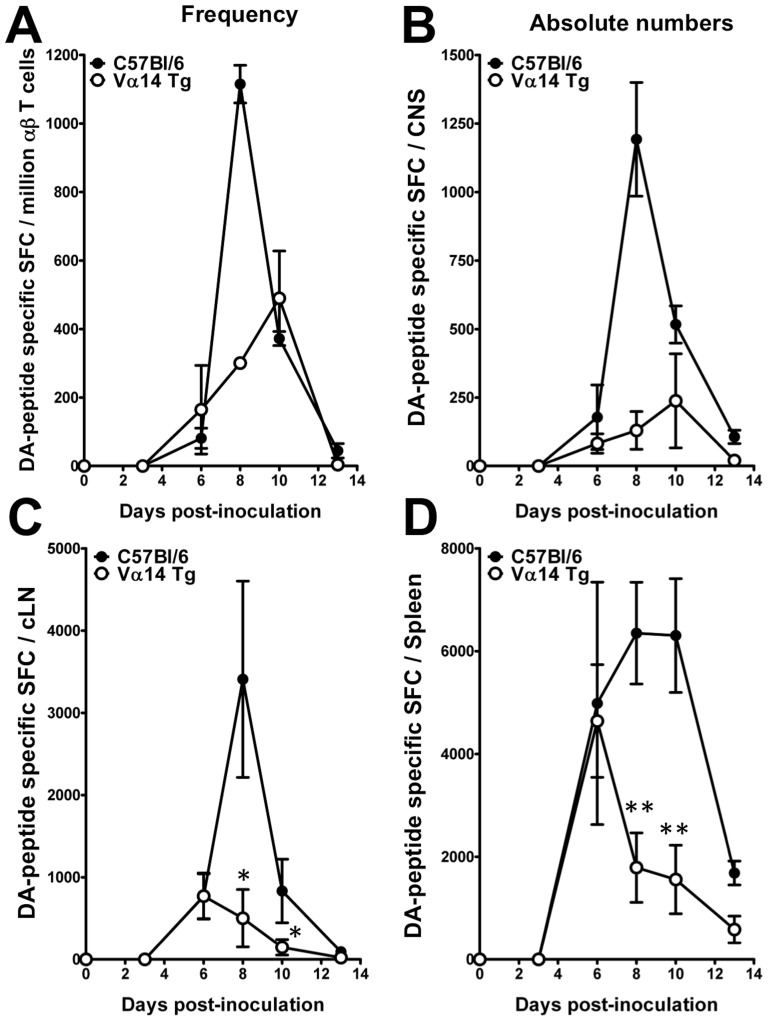
Delayed and blunted VP2_122–130_ specific CD8 T cell response in TMEV-infected TCR Vα14 Tg mice. (A) Frequency and (B–D) absolute number of the VP2_122–130_ specific CD8 T cells in the CNS and secondary lymphoid organs of Vα14 Tg C57Bl/6 mice and C57Bl/6 littermates prior to (day 0) or 3, 6, 8, 10, or 13 days after infection with 10^4^ pfu of TMEV. The magnitude of the TMEV-specific CD8 T cell response was determined by *ex vivo* IFN-γ ELISpot. To this end, mononuclear cells were restimulated or not with 10 µM of the H2-D^b^-restricted VP2_122–130_ epitope and IFN-γ spot-forming cells (SFC) were enumerated. (A–B) Representation of the VP2_122–130_ specific CD8 T cell response among percoll purified mononuclear cells of the CNS. Each individual timepoint represents 2 independent experiments on pooled mononuclear cells purified from 3–4 mice/group. Data represent the mean ± SEM. (C–D) Representation of the VP2_122–130_ specific CD8 T cell response in the cervical lymph nodes (C) and spleen (D). Data represents two individual experiments in which the organs of 6–8 mice/group and per timepoint were analysed individually. Data represent the mean of *n* individual mice ± SEM.

In C57Bl/6 mice TMEV infection is fully cleared by an anti-viral CD8 T cell response that essentially targets the immunodominant, H2-D^b^ restricted epitope VP2_122–130_ [42]. Inhibition of this anti-viral CD8 T cell response in the Vα14 Tg mice could contribute to the defect in TMEV elimination. To test this hypothesis we enumerated TMEV-specific CD8 T cells using an IFN-γ ELIspot assay. Figure 5 reveals the frequency (A) and absolute number (B) of effector CD8 T cells that responded to VP2_122–130_ in the CNS of TMEV infected mice. These data indicate that a sizeable anti-viral CD8 T cell response infiltrates the CNS of both C57Bl/6 wild-type mice and Vα14 Tg mice. However, the accumulation of TMEV-specific CD8 T cells is delayed in the CNS of TMEV infected Vα14 Tg mice (peak at day 10) as compared to infected wild-type controls (peak at day 8) (Fig 5A). In addition, the magnitude of the TMEV-specific CD8 T cell response is reduced in the CNS of Vα14 Tg mice compared to infected wild-type mice (Fig 5B). At the peak of CD8 T cell infiltration in the Vα14 Tg mice the absolute numbers of TMEV specific CD8 T cells amounts to 130+/−69, which is 9-fold less than that observed in wild-type mice where the TMEV-specific CD8 T cell response amounted to 1192+/−207. The blunting of the CD8 T cell response is likely to originate in the secondary lymphoid organs. Indeed, the mice enriched in iNKT cells mount a CD8 T cell response that is indistinguishable from that of infected wild-type mice 6 days post-infection in the cervical lymph nodes (Fig 5C) and the spleen (Fig 5D). However, in wild-type mice the TMEV-specific CD8 T cells response expands until day 8–10, while the TMEV-specific CD8 T cell response in the Vα14 Tg mice declines from day 6 onwards. These observations indicate that the increased viral load observed in the CNS of TMEV-infected Vα14 Tg mice correlates with a delayed recruitment of a numerically reduced antiviral CD8 T cell response to the CNS.

### CNS infiltrating iNKT cells express a pro-inflammatory gene-expression profile

**Figure 6:**
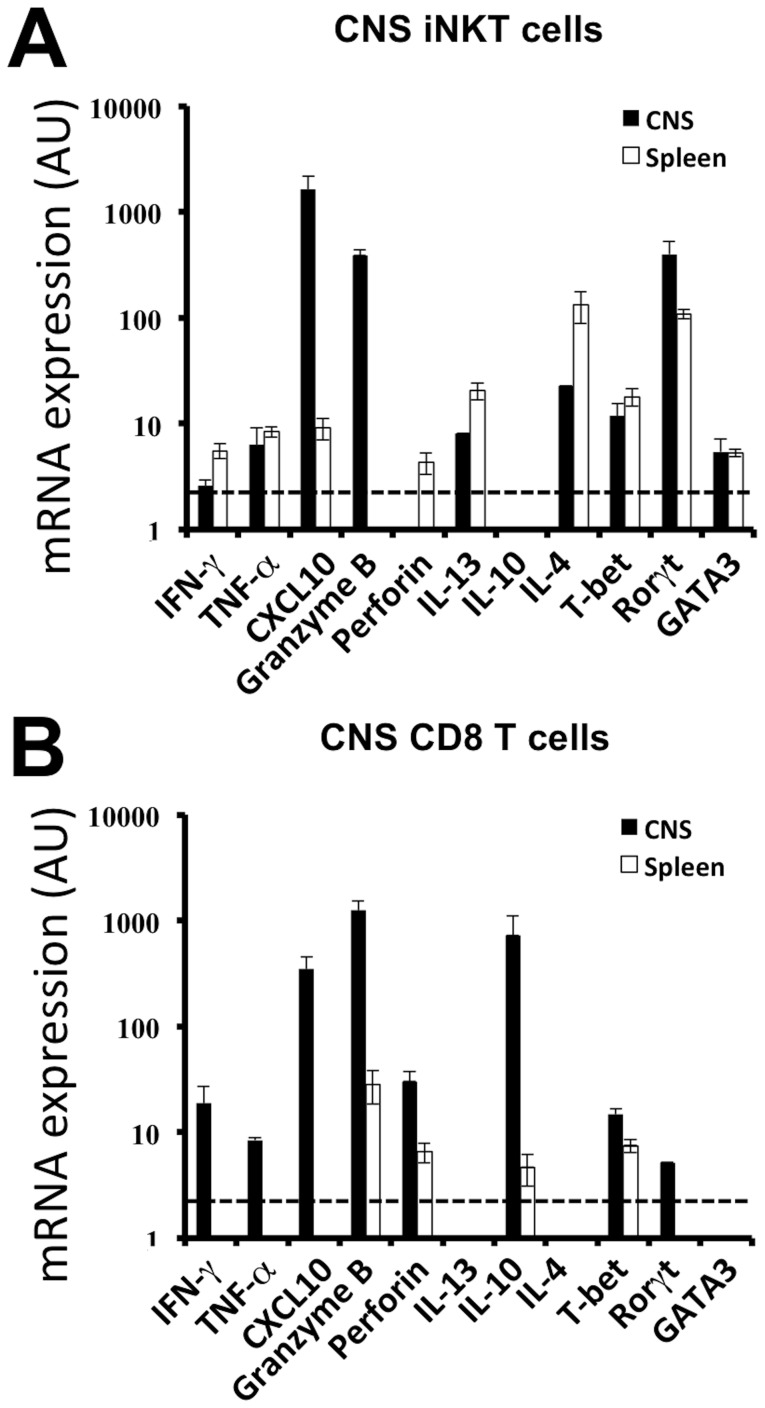
iNKT cells and CD8 T cells in the CNS of TMEV-infected mice express a cytotoxic phenotype. CD1d:α-GalCer tetramer^+^ TCRβ^+^ iNKT cells (A) and TCRβ^+^ CD8^+^CD4^−^ (B) T cells were FACS purified from the CNS of Vα14 Tg mice 8 days after TMEV infection. Real-time PCR analysis was performed on mRNA from purified iNKT cells and CD8 T cells from the spleen (white bars) and CNS (black bars). Cells were purified (black bars) from CNS-infiltrating mononuclear cells of 6–8 pooled Vα14 Tg mice or (white bars) from individual spleens. Data represent the mean ± SD of 2 individual experiments (CNS) or the mean of 4 individual mice ± SD (spleen).

To further understand the relative contribution of iNKT cells to the observed phenotype we electronically isolated α-GalCer:CD1d tetramers positive iNKT cells from the CNS and spleen of the Vα14 Tg mice, using a previously described protocol [56], 8 days post infection with TMEV. This timepoint corresponds to the peak of iNKT cell infiltration in the CNS of Vα14 Tg mice (Fig 2C). CD8 T cells were isolated in parallel. Organs from at least 4 mice were pooled, in each of the 2 experiments gene expression of a number of nuclear factors and signature cytokines was analysed by real-time qPCR. The gene-expression profile of CNS-infiltrating iNKT cells differs from that of splenic iNKT cells (Fig 6A). Relative to its splenic counterparts CNS iNKT cells revealed a strongly increased expression of *granzyme B,* and *CXCL10* and reduced transcription of *il4* and *il13*. This cytokine profile is consistent with a proinflammatory role of iNKT cells in the CNS during viral infection. When assessing the gene-expression of the nuclear factors T-bet, GATA3, and Rorγt we observed that *tbet* and *gata3* expression was comparable but the intensity of *ror*γ*t* message had increased among CNS infiltrating iNKT cells. The CD8 T cells, notably in the CNS, expressed an anticipated inflammatory and cytotoxic profile associated with the expression of *granzyme B, IFN*γ, *perforin, CXCL10, and tbet* (Fig 6B). Strong IL-10 message was equally detected among CD8 T cells, which is known to tune the effector CD8 T cell response to avoid excessive tissue damage [15], [57]. These data indicate that after TMEV infection the phenotype of iNKT cells differs drastically between the spleen and the CNS. In the spleen, where a blunting of the CD8 T cell response is observed, the iNKT cells express a phenotype with pronounced expression of *il4* and *il13*. In the CNS the infiltrating iNKT cells express higher levels of pro-inflammatory or even cytotoxic genes, implying a pro-inflammatory phenotype of iNKT cells locally at the site of viral infection.

### Treatment with exogenous iNKT cell ligands inhibits the TMEV-specific CD8 T cell response

**Figure 7:**
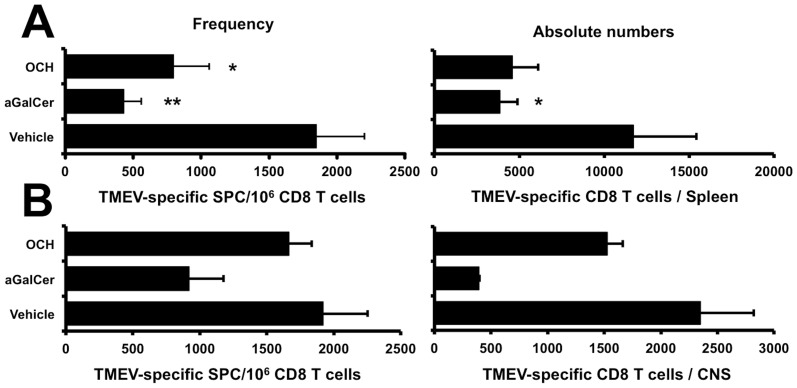
Activation of iNKT cells inhibits the VP2_122–130_-specific CD8 T cell response in TMEV infected C57Bl/6 mice. Frequency (left) and absolute number (right) of the VP2_122–130_-specific CD8 T cells in the spleen (A) and CNS (B) of C57Bl/6 mice 8 or 9 days after infection with 10^4^ pfu of TMEV. On the day of infection mice received a single injection of 4 µg of α-GalCer, OCH, or vehicle *i.p*. The magnitude of the TMEV-specific CD8 T cell response was determined by *ex vivo* IFN-γ ELISpot. To this end, mononuclear cells were restimulated or not with 10 µM of the H2-D^b^-restricted VP2_122–130_ epitope and IFN-γ spot-forming cells (SFC) were enumerated. (A) Data represents two individual experiments in which the organs of 4 mice/group were analysed individually. The mean of 8 individual mice ± SEM is presented. (B) Represents 2 independent experiments on pooled mononuclear cells purified from the CNS of 4 mice/group. Data represent the mean ± SEM.

We next assessed whether the activation of iNKT cells in wild-type C57Bl/6 mice would influence the magnitude of the TMEV-specific CD8 T cell response upon infection. To this end C57Bl/6 mice were injected *i.p.* on the day of TMEV infection with 4 µg of α-galactosylceramide (α-GalCer) or OCH, an analogue with a truncated sphingosine chain. Injection of the iNKT cell agonists reduced the frequency and absolute numbers of TMEV-specific CD8 T cells in the spleen compared to vehicle treated mice (Fig 7A). This demonstrates that the activation of iNKT cells by α-GalCer or OCH can restrain the mounting CD8 T cell response. This inhibition of the peripheral immune response impacted on the magnitude of the CD8 T cell response in the CNS (Fig 7B). In the α-GalCer treated mice a less pronounced accumulation of CD8 T cells was observed in the CNS both in terms of frequency and absolute numbers. In OCH treated mice the impact on the TMEV-specific CD8 T cell response in the CNS was less pronounced as compared to vehicle treated mice (Fig 7B). No mortality was observed, and no variation in TMEV transcripts could be detected in α-GalCer, OCH, or vehicle injected mice (data not shown). These data indicate that the activation of iNKT cells using exogenous glycolipid antigens can restrain the magnitude of the TMEV-specific CD8 T cell response in WT resistant C57Bl/6 mice without significantly affecting the course of the infection.

## Discussion

In the CNS microglial cells and reactive astrocytes express CD1d in inflammatory lesions indicating their capacity to present antigen to iNKT cells [58]. In addition, the CNS is rich in lipids and glycolipids that include self-antigens for iNKT cells [59]. Infection of the CNS with the neurotropic TMEV provokes immune intervention including the local accumulation of iNKT cells. In resistant C57Bl/6 mice the accumulation of iNKT cells peaks 8 days after infection. This coincides with the peak of the virus-specific CD8 T cell response in the CNS, which is sufficient to clear infection in resistant H2-D^b^ strain. At this timepoint in the CNS the CD8 T cell response targeting the immunodominant VP2_122–130_ epitope mounted to 1 250 cells, as assessed by IFNγ Elispot. At the same time on average 45 000 iNKT cells were found within the same infiltrate underscoring the efficiency of iNKT cells to accumulate in the CNS during viral infection. The detection of iNKT cells in the CNS prior to inflammation is likely to contribute to this increased efficacy. We could establish that iNKT cells represent 10% of CNS-resident T cells amounting to 4 000 iNKT cells per mouse CNS. This is a small, but significant number given their highly biased TCR repertoire with common antigen specificity. Two arguments strongly imply that this was not due to blood contamination. First, all mice were transcardially perfused prior to the dissection of the CNS. Second, when comparing the frequency of iNKT cells among circulating T cells to those isolated from the CNS a significantly increased proportion of iNKT was observed in the CNS. These observations strongly argue that in the absence of inflammation, iNKT cells contribute to the surveillance of different organs and tissues, including those that benefit from immune privilege. Due to their low abundance in the CNS and the technical difficulties associated with the use of tetramers for immunohistological analyses we could not determine the exact location of the iNKT cells within the CNS. In man, the CSF contains only few immune cells (1–5/ µl). These cells are mainly central memory CD4^+^ T cells implying that memory T cells can extravasate from the blood into the CSF, even in the absence of inflammation [60], [61]. As iNKT cells are innate memory cells expressing a broad chemokine/homing receptor profile for non-lymphoid tissues this could, similarly, permit their migration across the blood-CSF barriers [53]–[55]. This is further emphasised by the expression of CCR6, a homing receptor permitting the entry into the CNS via the choroid plexus [62], which can be detected on human CD8^+^ and CD4^−^CD8^−^ iNKT cells [53].

In the Vα14 Tg mice that are enriched in iNKT cells 38% of mice developed a fatal acute encephalomyelitis, which was associated with a failure to clear TMEV infection. This was associated with a marked blunting of the anti-viral CD8 T cell response. This resulted in a delay in the recruitment of the anti-viral CD8 T cell response to the CNS of Vα14 Tg mice (peak at day 10) when compared to wild-type mice (peak day 8). In addition, the magnitude of the CNS infiltrating VP2_122–130_-specific CD8 T cell response was reduced by 5-fold in the Vα14 Tg mice compared to the wild-type mice. This deficit originated in the secondary lymphoid organs. Intriguingly, the priming of the anti-viral CD8 T cell response remained unaltered between the Vα14 Tg mice and the TMEV infected wild-type controls. However, from day 6 onwards the VP2_122–130_-specific CD8 T cell response was strongly blunted in the Vα14 Tg mice. We did not address the underlying mechanisms directly. However, we have previously used the Vα14 Tg mice to study the impact of iNKT cells on experimental autoimmune encephalomyelitis (EAE). In this model, the MOG_35–55_-specific CD4^+^ T cell response was regulated after egress from the draining lymph nodes. Using this model we could exclude deficits in the T cell repertoire [56], exclude a role for IL-4 [38], and exclude the requirement for CD1d to impose iNKT cell immune regulation [56]. Collectively, these data indicate that, in the Vα14 Tg mice, iNKT cells can actively regulate an anti-viral CD8 T cell response via as yet unidentified mechanisms.

Two non-mutually exclusive mechanisms are likely to explain the mortality observed in the TMEV-infected Vα14 Tg mice. One mechanism would argue that the absence of a TMEV-specific CD8 T cell response permits uninhibited viral dissemination. Virus driven tissue damage would then prove fatal over time. This scenario is supported by the persistence of TMEV over time, the blunted CD8 T cell response in the CNS and the fact that both the CD8-deficient H2-D^b-/-^ and β2-microglobulin^-/-^ mice that fail to clear TMEV infection succumb to a fatal outcome in a similar percentage of mice [63]–[66]. However, a few arguments are available to suspect a more complex mechanism. First, the CD8 T cell response is inhibited but not absent from the Vα14 Tg mice. Furthermore, the TMEV viral load in the Vα14 Tg mice at late stages is inferior to that seen in the H2-D^b-/-^ mice [63], [64]. This indicates that the Vα14 Tg mice still partly control TMEV infection. In this case the mortality is unlikely to be solely due to viral mediated tissue damage. This is consistent with the use of the less aggressive DA strain of TMEV which differs from the GDVII strain by a more moderate neurolytic activity [41]. A second scenario would imply that the mortality of the Vα14 Tg mice is potentially driven by an excessive encephalitogenic immune response. This response is relatively poor in anti-viral CD8 T cells but rich in iNKT cells (4.5 10^5^ iNKT cells at day 8 *p.i.*). The suggestion that this iNKT cell response could contribute to neural damage is supported by our quantitative PCR experiments on electronically purified iNKT cells from the CNS of TMEV-infected Vα14 Tg mice. These purified CNS iNKT cells expressed little IL-4, IL-10 and IL-13 transcripts, but are strong producers of Granzyme B and CXCL-10, indicative of a pro-inflammatory cytotoxic function. In concordance with this scenario, when we infected H2-D^b -/-^ + H2-K^b -/-^ (n = 21) and compared them to infected H2-D^b -/-^ + H2-K^b -/-^ + β2-microglobulin^-/-^ (n = 16) we observed an increased mortality (14% vs 69%; p = 0.002) and viral load at 45 days p.i. (0.3+/−0.1 (n = 16) vs 1.4+/−0.4 (n = 5) TMEV/HPRT) among surviving H2-D^b -/-^ + H2-K^b -/-^ + β2-microglobulin^-/-^ mice. This underlines that non-classical MHC I molecules, which are absent in mice deficient for β2-microglobulin, are non-redundant for the protection against CNS viral infections. Similarly, an immuno-pathological event during the acute encephalomyelitis probably explains the difference of mortality of B10.S mice bearing different *Il22* variants after intracerebral TMEV inoculation [49].

Taken together, our observations indicate that iNKT cells are present in the CNS even in the absence of inflammation. The magnitude of CNS iNKT cells dramatically increases after local virus infection. This implies that iNKT cells may play an important role in the immune surveillance in the CNS. In addition we demonstrated that the enrichment of iNKT cells regulates the CD8 T cell response after virus infection. The fact that this could be reproduced by injecting α-GalCer or OCH might raise some caution for strategies that aim to improve vaccination efficacy with iNKT cell ligands.
